# TPPU enhanced exercise‐induced epoxyeicosatrienoic acid concentrations to exert cardioprotection in mice after myocardial infarction

**DOI:** 10.1111/jcmm.13412

**Published:** 2017-12-19

**Authors:** Yuan Guo, Fei Luo, Xv Zhang, Jingyuan Chen, Li Shen, Yi Zhu, Danyan Xu

**Affiliations:** ^1^ Department of Cardiovascular Medicine The Second Xiangya Hospital Central South University Changsha Hunan China; ^2^ Department of Physiology and Pathophysiology Collaborative Innovation Center of Tianjin for Medical Epigenetics Tianjin Medical University Tianjin China

**Keywords:** myocardial infarction, exercise, soluble epoxide hydrolase inhibitor, endothelial progenitor cells, angiogenesis, microRNA‐126

## Abstract

Exercise training (ET) is a safe and efficacious therapeutic approach for myocardial infarction (MI). Given the numerous benefits of exercise, exercise‐induced mediators may be promising treatment targets for MI. C57BL/6 mice were fed 1‐trifluoromethoxyphenyl‐3‐(1‐propionylpiperidine‐4‐yl) urea (TPPU), a novel soluble epoxide hydrolase inhibitor (sEHI), to increase epoxyeicosatrienoic acid (EET) levels, for 1 week before undergoing MI surgery. After 1‐week recovery, the mice followed a prescribed exercise programme. Bone marrow‐derived endothelial progenitor cells (EPCs) were isolated from the mice after 4 weeks of exercise and cultured for 7 days. Angiogenesis around the ischaemic area, EPC functions, and the expression of microRNA‐126 (miR‐126) and its target gene *Spred1* were measured. The results were confirmed *in vitro* by adding TPPU to EPC culture medium. ET significantly increased serum EET levels and promoted angiogenesis after MI. TPPU enhanced the effects of ET to reduce the infarct area and improve cardiac function after MI. ET increased EPC function and miR‐126 expression, which were further enhanced by TPPU, while *Spred1* expression was significantly down‐regulated. Additionally, the protein kinase B/glycogen synthase kinase 3β (AKT/GSK3β) signalling pathway was activated after the administration of TPPU. EETs are a potential mediator of exercise‐induced cardioprotection in mice after MI. TPPU enhances exercise‐induced cardiac recovery in mice after MI by increasing EET levels and promoting angiogenesis around the ischaemic area.

## Introduction

Myocardial infarction (MI) remains a leading cause of mortality and morbidity worldwide despite a growing number of pharmacological interventions. Exercise training (ET)‐based cardiac rehabilitation is a new and emerging therapeutic approach that improves the prognosis of MI 1, 2. Although exercise‐induced cardioprotective effects have been widely studied in recent years, the mechanisms of its benefits are still not fully clarified, and the enrolment of cardiac rehabilitation is generally low worldwide.

Angiogenesis, the formation of new capillaries from pre‐existing vessels, plays an essential role in cardiac repair after MI and is responsible for restoring blood supply to the ischaemic myocardium and for preserving cardiac function 3. It has been well documented that endothelial progenitor cells (EPCs) promote angiogenesis around the ischaemic area in infarcted hearts 4. Previously, we found that exercise markedly induced EPC function in mice, which was consistent with existing data 5, 6. However, the mechanism of exercise‐improved EPC function is comprehensive and poorly studied.

When metabolized by P450 enzymes, arachidonic acid (AA) generates four regioisomeric epoxyeicosatrienoic acids (EETs): 5,6‐EET, 8,9‐EET, 11,12‐EET and 14,15‐EET 7. Of these four EETs, 14,15‐EET is mainly produced in the heart. EETs function as autocrine and paracrine effectors in the cardiovascular system and have multiple cardioprotective properties 8. However, EETs are unstable and easily metabolized by soluble epoxide hydrolase (sEH) to form the corresponding dihydroxyeicosatrienoic acids (DHETs), which have weak biological activity. Accordingly, sEH inhibitors (sEHIs) are widely reported as pre‐clinical agents for treating cardiovascular diseases by increasing EET concentrations 8. 1‐Trifluoromethoxyphenyl‐3‐(1‐propionylpiperidine‐4‐yl) urea (TPPU), a novel sEHI, has more potent cardioprotective and pharmacological function than earlier sEHIs 9, 10. Interestingly, our group has found that sEHI significantly improve EPC function in patients after MI by increasing EET concentrations 11. However, whether exercise and sEHI can induce EETs and whether EETs can further enhance exercise‐induced EPC functions after MI have not been reported.

MicroRNAs (miRNAs) consist of about 22 nucleotides and are a class of small non‐coding RNAs that regulate gene expression by targeting mRNAs for cleavage or translational repression 12. Several miRNAs are closely related to angiogenesis and are referred to as angiomiRs 13. The angiomiR miR‐126 has been well demonstrated as being associated with angiogenesis after MI 14. MiR‐126 exerts its function by modulating a cluster of target genes, which includes *VEGFA, PIK3R2, vascular cell adhesion molecule 1* (*VCAM1)* and sprouty‐related EVH1 domain containing 1 (*SPRED1*) 15, 16. Of these genes, there is much evidence that *SPRED1* is a target of miR‐126 in promoting angiogenesis 17. Interestingly, recent studies have not only revealed important roles for miR‐126 in response to cardiac ischaemia, but have also demonstrated that exercise up‐regulates miR‐126 expression 12, 18, 19, 20, 21. Accordingly, we speculated that miR‐126 may up‐regulate exercise‐induced EPC function in mice after MI.

Protein kinase B/glycogen synthase kinase 3β (AKT/GSK3β) is a critical cardioprotective signalling pathway against cardiac injury after myocardial ischaemia and is activated by ischaemia, exercise, and many other factors 22. Previously, we demonstrated the activation of the AKT/GSK3β pathway in bone marrow‐derived EPCs in MI mice after 4 weeks of exercise 5. Furthermore, it has been widely reported that the AKT/GSK3β signalling pathway regulates miR‐126 expression and thereby promotes angiogenesis after MI 23. Moreover, sEHIs also exert their protective actions by activating the AKT/GSK3β pathway 24. Consequently, we believe that EETs can promote and enhance exercise‐induced miR‐126 expression by stimulating the AKT/GSK3β pathway.

Based on the above hypothesis, we designed this study in MI mice to demonstrate that exercise promotes EPC functions and cardiac repair after MI by increasing EET concentrations. Moreover, we examined whether TPPU can enhance the exercise‐induced cardioprotection and thereby provide more evidence for the therapeutic function of sEHIs.

## Materials and methods

### MI model and ET protocol

As with our previous work, a MI model was constructed using male C57BL/6 mice (6 weeks old, obtained from the Medical Experimental Animal Center of Hunan Province, China) by ligating the left anterior descending coronary artery. Mice that underwent sham surgery (non‐MI mice), where the coronary artery was not ligated, were used as the control 5, 25. Post‐surgery, the mice recovered for 7 days and then were exercised following a progressively increased workload programme prescription. Exercise protocol began with 10 min. at a speed of 10 m per minute and added 10 min. per day with a speed increased 1 m per minute. After 5 days exercise, all mice had 2‐day rest. From the second week, exercise mice underwent ET following a protocol of 1 hr at a speed of 15 m per minute and exercised 5 days per week 10. The protocol was approved by the Animal Research Committee, Central South University, Hunan, China.

### Drug delivery

The potent and selective TPPU was synthesized in the laboratory of Prof. Bruce D. Hammock (UC Davis, USA) 26. Compared with earlier sEHIs, TPPU has improved water solubility and high oral availability such that it can be delivered in drinking water. TPPU stock solution was prepared by dissolving in polyethylene glycol 400 (PEG400; Sigma‐Aldrich, St. Louis, MO, USA) at a final concentration of 0.2%; 15 mg/l TPPU yielded a clear and stable stock solution at room temperature 10. For treatment, the stock solution was diluted to 0, 0.2, 1 and 5 mg/l.

### Experimental design

For the *in vivo* study, a MI mouse model was established as described above. After 1‐week recovery, the surviving mice were randomly divided into four groups: non‐myocardial infarction and non‐exercise training (NMI NET), non‐myocardial infarction with exercise training (NMI ET), myocardial infarction and non‐exercise training (MI NET), and myocardial infarction with exercise training (MI ET) (*n* = 18 each in five independent experiments, respectively. The survival rate of MI surgery is more than 80%). Mice in the ET groups underwent 4 weeks of exercise. Bone marrow‐derived EPCs were isolated and cultured for 7 days to test the relative expression of miR‐126. At the same time, serum 14, 15‐EET was measured.

To test the effect of TPPU on exercised mice, mice in the MI ET group were fed 0, 0.2, 1 or 5 mg/l TPPU for 1 week prior to the MI surgery and subsequent 4‐week exercise programme (*n* = 18 each in five independent experiments, respectively. The survival rate of MI surgery is more than 80%. The following survival rate was over 90% during exercise processing, and at least 12 mice in each group were studied. The mice were fed TPPU throughout the experiment. Bone marrow‐derived EPCs were isolated from the mice and cultured for 7 days. EPC functions and relative expression of miR‐126 and its target gene *Spred1* and the related signalling pathways were tested. At the same time, infarct area and cardiac function were measured.

For the *in vitro* study, a MI mouse model was established as above and the mice were exercised for 4 weeks after MI surgery for 1 week as described above (MI ET group). Bone marrow‐derived EPCs were isolated from the group and cultured for 7 days, and were treated with 0, 0.1, 1 and 10 μM TPPU, or 10 μM TPPU plus 0.5 μM 14,15‐EEZE, a 14,15‐EET–specific antagonist, for 24 hrs. The relative expression of miR‐126 and the related signalling pathways were measured.

### Infarct area and capillary density

For histological analysis, mouse hearts were sheared at the end of the experiment, rinsed three times with phosphate‐buffered saline (PBS) and fixed with 4% paraformaldehyde. After paraffin‐embedding, Masson's trichrome staining was performed to identify the infarct area, which was expressed as a percentage of the total LV size.

Capillary density was determined by immunohistochemical staining with CD31 antibody. Immunohistochemical staining was performed using the two‐step immunohistochemical technique with diaminobenzidine (DAB; Sigma‐Aldrich, St. Louis, MO, USA) as described in the manufacturer's instructions. After restaining with hematine, the samples were coverslipped and photographed. The capillaries around the ischaemic area were counted as the mean of five randomly selected fields under ×400 magnification.

### Cardiac function

At the end of the experiment, the LV function variables were assessed by transthoracic echocardiography, which was performed using a 15‐MHz phased array transducer (Siemens ACUSON S3000). Left ventricular (LV) dimensions were examined digitally in M‐mode tracings and averaged from three consecutive cardiac cycles. LV end‐systolic diameter (LVESD) and end‐diastolic diameter (LVEDD), interventricular septal thickness in diastole and LV posterior wall thickness were assessed. The percentage of LV fractional shortening (FS) was calculated as follows: FS% = (LVEDD‐LVESD)/LVEDD x 100 (%). Cardiac function was also measured as the ejection fraction EF (%) = (EDV – ESV)/EDV × 100%, where EDV and ESV are the end‐diastolic and end‐systolic left ventricular internal dimension (LVID), respectively. All measurements were carried out by three experienced technicians who were unaware of the experimental design.

### EET concentration assay

14,15‐EET was measured by liquid chromatography coupled with tandem mass spectrometry (LC‐MS/MS, ACQUITY UPLC) in the laboratory of Prof. Yi Zhu at Tianjin Medical University as they previously described 27, 28.

### Bone marrow‐derived EPC isolation and culture

After 4 weeks of exercise, bone marrow‐derived EPCs were isolated and cultured by density gradient centrifugation as described in our previous work 5.

### Migratory ability assay

Migratory ability of the EPCs was measured as we described previously 5. Besides, scratch wound healing assay was also carried out to estimate the migratory ability of EPCs. After 7 days of cultivation, EPC monolayers were scraped with a sterile pipette tip to generate a cell‐free zone. The cells were then washed with medium and cultured for another 24 hrs. Photographs were taken at 0 and 24 hrs under ×100 magnification. The results were the mean of five random fields.

### Proliferative ability and *in vitro* angiogenesis assay

After 7 days of culture, the proliferative ability and *in vitro* angiogenesis abilities of the EPCs were assayed as we described previously 5. A capillary tube was defined as a tubular structure whose length was four times as long as its width and was analysed by ImageJ. The results were the mean number of capillary tubes in five random fields counted under ×200 magnification.

### RNA extraction and quantitative real‐time PCR (qPCR)

Total RNA from 1 × 10^6^ cells was harvested using TRIzol (Invitrogen, Carlsbad, CA, USA) according to the manufacturer's instructions. Total RNA (800 ng) was reverse‐transcribed with a TaqMan MicroRNA Reverse Transcription Kit. The miRNA qPCR assay was performed using TaqMan microRNA assays and the TaqMan Universal Master Mix II, no UNG, on a 7300 qPCR system (all from Applied Biosystems, Foster, CA, USA). For the mRNA qPCR assay, complementary DNA (cDNA) was generated using a RevertAid First Strand cDNA Synthesis Kit (Thermo Scientific, Carlsbad, CA, USA) and was performed with gene‐specific primers and SYBR Green Master Mix (Applied Biosystems). The data were normalized to a single reference gene (miRNA, *U6*; mRNA, *GAPDH*). The *Mir126* and *U6* primer sequences are available upon request (Life Technologies, Carlsbad, CA, USA). The *Spred1* and *GAPDH* primer sequences are as follows:

**Table:** 

	Sequences
*Spred1*	
Sense	F5′‐TACCTTTCAGAGTCCTGCTGATG‐3′
Antisense	R5′‐GGGAAGTGTCTTCTTCAGTCGT‐3′
*GAPDH*	
Sense	F5′‐AGGTCGGTGTGAACGGATT‐3′
Antisense	R5′‐AATCTCCACTTTGCCACTGC‐3′

Relative gene expression was quantitated using the comparative threshold cycle value (∆CT) method with the above primers. The relative gene expression was calculated as fold change = 2^−∆(∆Ct)^.

### Western blotting

As described in our previous work, Western blotting was performed with rabbit antimouse AKT, phosphorylated AKT (p‐AKT), GSK3β and p‐GSK3β antibodies (Cell Signaling Technology, Danvers, MA, USA); mouse antimouse SPRED1 (Abcam, Cambridge, MA, USA) and β‐actin were used as loading controls 5. ImageJ (NIH, Bethesda, MD, USA) was used to quantify the pixel intensities of immunoreactive bands.

### Statistical analysis

All experiments were performed in duplicate three to five times. Results are expressed as the mean ± S.E.M. Comparison among experimental groups of NMI NET, NMI ET, MI NET and MI ET was performed using two‐way analysis of variance (ANOVA). Comparison among TPPU‐treated experimental groups was performed using one‐way ANOVA. Tukey's multiple comparisons test was used to compare the simple effect between groups. *P *<* *0.05 was considered statistically significant.

## Results

### TPPU increased exercise‐induced effects on inhibiting cardiac enlargement and improving cardiac function by increasing EET levels in MI mice

**Figure 1:**
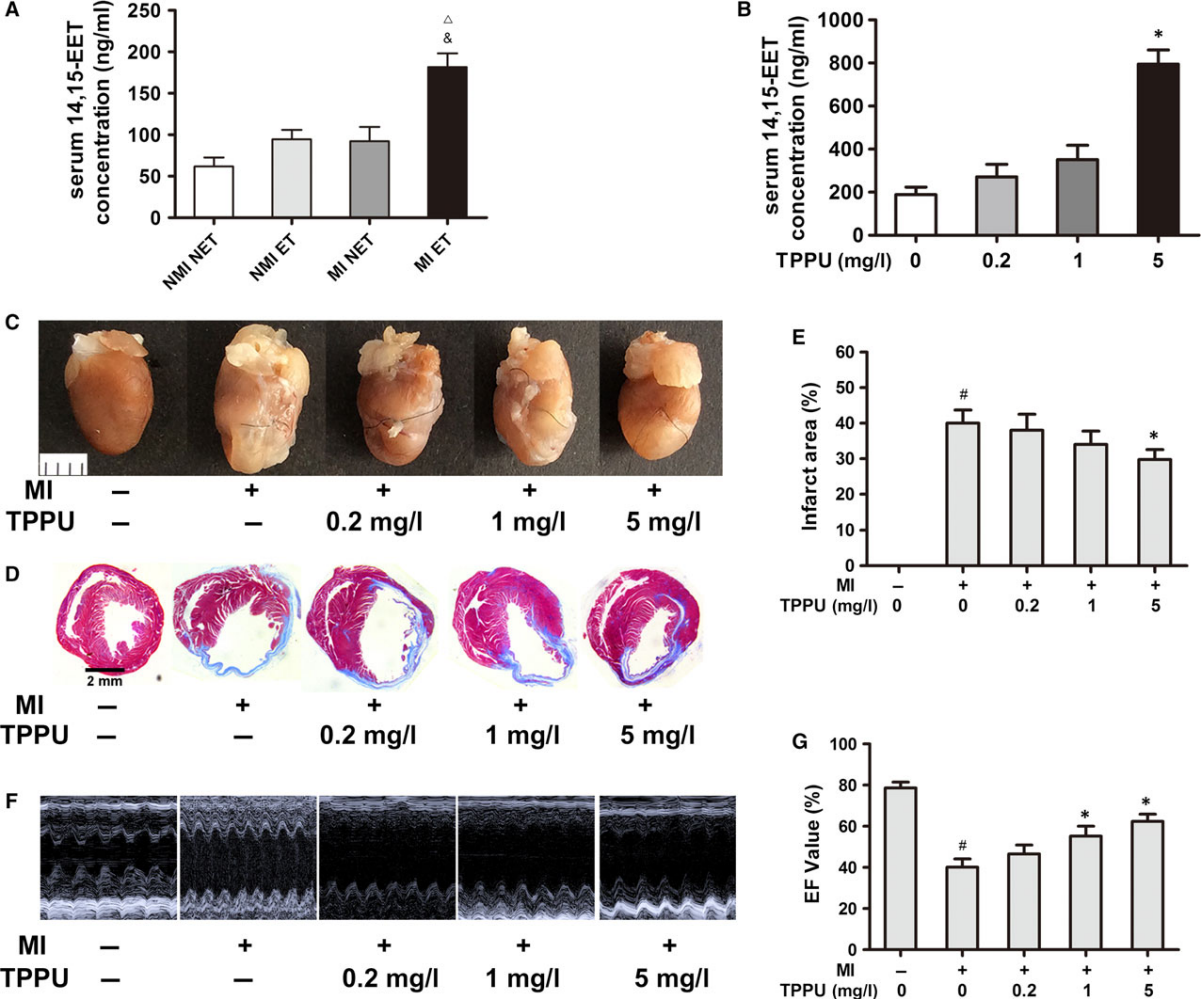
TPPU inhibited cardiac enlargement and improved cardiac function by increasing EET levels in MI mice. MI or non‐MI mice were exercised for 4 weeks, and 200 μl of serum was obtained. Serum 14,15‐EET was measured by LC‐MS/MS (**A**). In the *in vivo* TPPU‐treated group, serum 14,15‐EET was measured by LC‐MS/MS (**B**). Infarcted heart size and infarct area following TPPU treatment and Masson's trichrome staining were compared (**C** and **D**). Quantitative analysis of infarct area (**E**). Left ventricular function was assessed by transthoracic echocardiography, and EF values were determined for cardiac function (**F**). Quantitative analysis of cardiac function (**G**). MI: myocardial infarction; EF: ejection fraction; EET, epoxyeicosatrienoic acid; NMI NET: non‐myocardial infarction and non‐exercise training; NMI ET: non‐myocardial infarction with exercise training; MI NET: myocardial infarction and non‐exercise training; MI ET: myocardial infarction with exercise training; TPPU: 1‐trifluoromethoxyphenyl‐3‐(1‐propionylpiperidine‐4‐yl) urea. ^▵^ *P *<* *0.05, *versus* NMI NET. ^&^ *P *<* *0.05, *versus* MI NET. ^#^ *P *<* *0.05, *versus* non‐MI groups treated with 0 mg/l TPPU. *P <* *0.05, *versus* MI ET groups treated with 0 mg/l TPPU (*n* = 5).

Previously, we demonstrated that 4‐week ET significantly inhibited cardiac enlargement by promoting EPC function in mice after MI 5. In this study, 4 weeks of exercise significantly increased serum 14,15‐EET concentrations to 181.5 ng/ml in MI ET mice, an increase of about 2.9‐fold and 2.0‐fold as compared with NMI NET mice and MI NET mice, respectively (all, *P *<* *0.05) (Fig. 1A). This indicates that exercise increases serum 14,15‐EET concentrations.

To test whether 14,15‐EET exerted cardioprotection after exercise in mice, we treated the mice with TPPU. In MI ET mice treated with 5 mg/l TPPU, serum 14,15‐EET concentrations were further increased to 794.8 ng/ml, confirming that TPPU is effective and significantly increases serum 14,15‐EET levels (*P *<* *0.05) (Fig. 1B).

MI surgery led to cardiac enlargement and reduced cardiac function, while TPPU significantly reversed this tendency in a dose‐dependent manner (Fig. 1C). Masson's trichrome staining showed a reduction of almost 10% in infarct size in MI ET mice after treatment with 5 mg/l TPPU when compared with MI ET mice treated with 0 mg/l TPPU (*P *<* *0.05) (Fig. 1D and E). MI significantly reduced cardiac function, and the EF and FS values were, respectively, decreased to 40.19% and 21.57% after 4 weeks of ischaemia (all, *P *<* *0.05). The MI ET mice treated with 5 mg/l TPPU had a significant increase in the two variables when compared with the MI ET mice treated with 0 mg/l TPPU (*P *<* *0.05) (Fig. 1F and G and Table 1).

**Table 1:** Ultrasonic echocardiography performance 4 weeks following MI surgery

Variables	NMI + 0 mg/l TPPU	MI + 0 mg/l TPPU	MI + 0.2 mg/l TPPU	MI + 1 mg/l TPPU	MI + 5 mg/l TPPU
HR (min^−1^)	367 ± 27	454 ± 23#	421 ± 25	400 ± 28*	375 ± 21*
LVEDD (mm)	3.61 ± 0.13	4.59 ± 0.14	4.42 ± 0.20	4.42 ± 0.17	4.15 ± 0.13
LVESD (mm)	2.18 ± 0.14	3.62 ± 0.15	3.33 ± 0.18	3.11 ± 0.19	2.71 ± 0.11
EDV (μl)	28.92 ± 0.92	34.64 ± 1.38	33.67 ± 1.81	32.85 ± 1.70	31.02 ± 1.01
ESV (μl)	6.14 ± 1.08	20.73 ± 1.13	18.0 ± 1.65	14.78 ± 1.31	11.57 ± 1.42
FS%	40.82 ± 3.36	21.57 ± 3.45#	25.12 ± 3.25	28.64 ± 3.71*	34.16 ± 3.18*
EF%	78.62 ± 3.83	40.19 ± 3.97#	46.54 ± 4.73	55.18 ± 5.30*	62.34 ± 3.31*

EF: ejection fraction; ESV: end‐systolic volume; EDV: end‐diastolic volume; FS: fractional shortening; HR: heart rate; MI: myocardial infarction; NMI: non‐myocardial infarction; LV: left ventricular; LVEDD: LV end‐diastolic diameter; LVESD: LV end‐systolic diameter. Data are expressed as the mean standard error of the mean (*n* = 5).

^#^ *P *<* *0.05, *versus* NMI group. *P <* *0.05, *versus* MI group.

### TPPU enhanced exercise‐induced effects on promoting angiogenesis and EPC functions in MI mice

**Figure 2:**
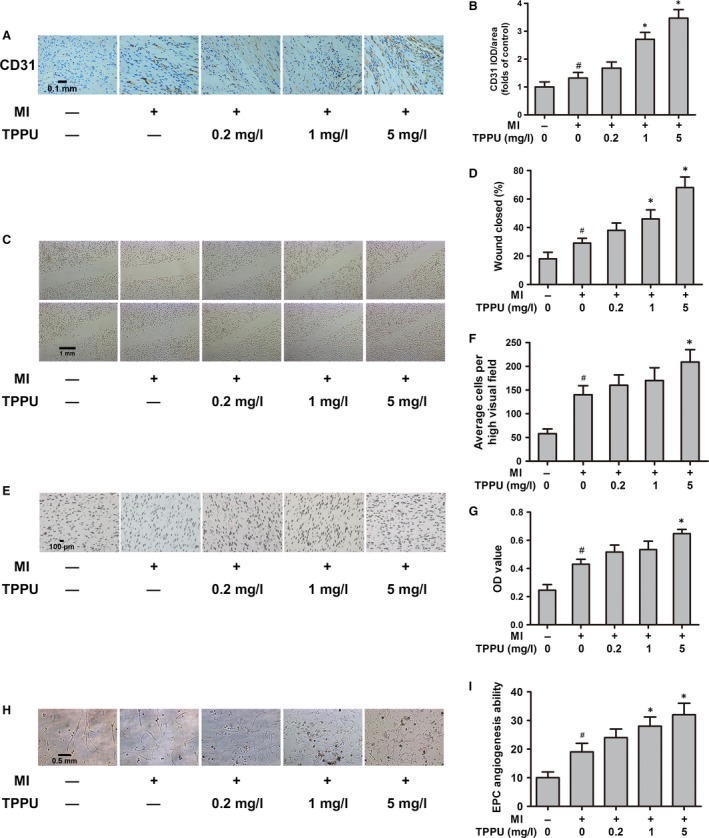
TPPU promoted angiogenesis around infarct area and EPC function in MI mice after exercise. Immunohistochemical staining for CD31 in the *in vivo* TPPU‐treated MI ET group (magnification ×400) (**A**). Quantitative analysis of CD31 positive rate (**B**). Scratch wound assay for identifying the proliferation of bone marrow‐derived endothelial progenitor cells (EPCs) from the TPPU‐treated MI ET group (×200 magnification) (**C**). Quantitative analysis of wound closure rate (**D**). Effect of TPPU on the EPC migratory ability (×200 magnification) (**E**). Quantitative analysis of EPC migratory ability (**F**). Effect of TPPU on the EPC proliferative ability that was measured by MTT method. The OD values of EPCs were measured under 490 nm light absorbance (**G**). The tube formation on Matrigel for identifying EPC *in vitro* angiogenesis ability (×100 magnification). A capillary tube was defined as a tubular structure whose length was four times as long as its width (**H**). Quantitative analysis of tube numbers (**I**). MI ET: myocardial infarction with exercise training; MI: myocardial infarction; TPPU: 1‐trifluoromethoxyphenyl‐3‐(1‐propionylpiperidine‐4‐yl) urea. ^#^ *P *<* *0.05, *versus* non‐MI groups treated with 0 mg/l TPPU. *P <* *0.05, *versus* MI ET groups treated with 0 mg/l TPPU (*n* = 5).

Both our previous study and existing data demonstrate that exercise promotes *in vitro* angiogenesis and EPC functions 5. Immunohistochemical staining was used to examine CD31 expression to determine the capillary density around the ischaemic area after TPPU intervention. The positive rate of CD31 was significantly increased after TPPU treatment, and the increase was dose‐dependent. In MI ET mice treated with 5 mg/l TPPU, the CD31‐positive rate was increased to 2.5‐fold, when compared with the MI ET mice treated with 0 mg/l TPPU (*P *<* *0.05) (Fig. 2A and B).

In addition, EPC functions, including proliferation, migration, and *in vitro* angiogenesis, were significantly enhanced after TPPU treatment, also in a dose‐dependent manner. Compared with 0 mg/l TPPU‐treated MI ET mice, 5 mg/l TPPU‐treated MI ET mice increased EPC migratory ability by 2.3‐fold and 1.5‐fold measured by scratch wound and transwell assay, respectively, proliferative ability by 1.5‐fold, and *in vitro* angiogenesis ability by 1.7‐fold (all, *P *<* *0.05) (Fig. 2C–I).

### TPPU up‐regulated miR‐126 expression in EPCs

**Figure 3:**
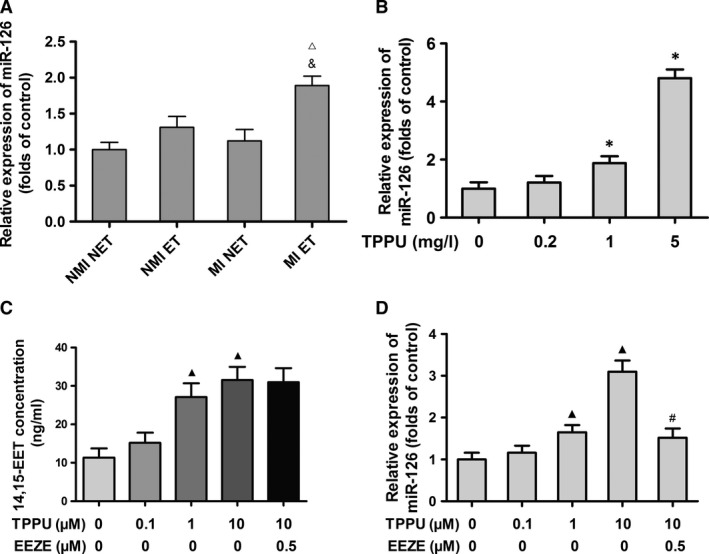
TPPU up‐regulated miR‐126 expression in endothelial progenitor cells (EPCs). Measurement of relative expression of miR‐126 in EPCs from myocardial infarction (MI) or non‐MI mice that were exercised for 4 weeks (**A**). Relative expression of miR‐126 in bone marrow‐derived EPCs from TPPU‐treated MI ET mice (**B**). LC‐MS/MS measurement of 14,15‐EET levels in 1 ml culture medium of TPPU‐treated and TPPU plus 14,15‐EEZE‐treated EPCs (**C**). Relative expression of miR‐126 in TPPU‐treated and TPPU plus 14,15‐EEZE‐treated EPCs (**D**). NMI NET: non‐myocardial infarction and non‐exercise training; NMI ET: non‐myocardial infarction with exercise training; MI NET: myocardial infarction and non‐exercise training; MI ET: myocardial infarction with exercise training; TPPU: 1‐trifluoromethoxyphenyl‐3‐(1‐propionylpiperidine‐4‐yl) urea. ^▵^ *P *<* *0.05, *versus* NMI NET. ^&^ *P *<* *0.05, *versus* MI NET. *P <* *0.05, *versus* MI ET groups treated with 0 mg/L TPPU. ^▲^ *P *<* *0.05, *versus* 0 μM TPPU group. ^#^ *P *<* *0.05, *versus* 10 μM TPPU group (*n* = 5).

The expression of miR‐126 in MI ET group was significantly higher than that in NMI NET group and the MI NET group, with 1.9‐fold and 1.7‐fold increase, respectively, when compared with the NMI NET group and the MI NET group (all, *P *<* *0.05) (Fig. 3A). Furthermore, TPPU further up‐regulated miR‐126 expression in a dose‐dependent manner. MI ET mice treated with 5 mg/l TPPU had 4.8‐fold up‐regulated miR‐126 expression compared with MI ET mice treated with 0 mg/l TPPU (*P *<* *0.05), indicating that TPPU further enhanced the exercise‐induced miR‐126 upregulation in EPCs (Fig. 3B).

To explore the effects of the sEHI on miR‐126 expression *in vitro*, we isolated and cultured the bone marrow‐derived EPCs from MI ET mice. After 7 days of culture, we treated the EPCs with 0, 0.1, 1, and 10 μM TPPU, or 10 μM TPPU plus 0.5 μM 14,15‐EEZE. We found that 14,15‐EET levels in the culture medium were significantly increased in tandem with TPPU concentration. 14,15‐EET levels were increased about 2.8‐fold after treatment with 10 μM TPPU when compared with treatment with 0 μM TPPU (*P *<* *0.05) (Fig. 3C). Consistently, 10 μM TPPU increased miR‐126 expression to 3.1‐fold, and the increase was also TPPU dose‐dependent (*P *<* *0.05). However, the effect of TPPU was significantly decreased by the addition of treatment with 0.5 μM 14,15‐EEZE (*P *<* *0.05) (Fig. 3D).

### Inhibiting miR‐126 promoted *Spred1* expression in EPCs

**Figure 4:**
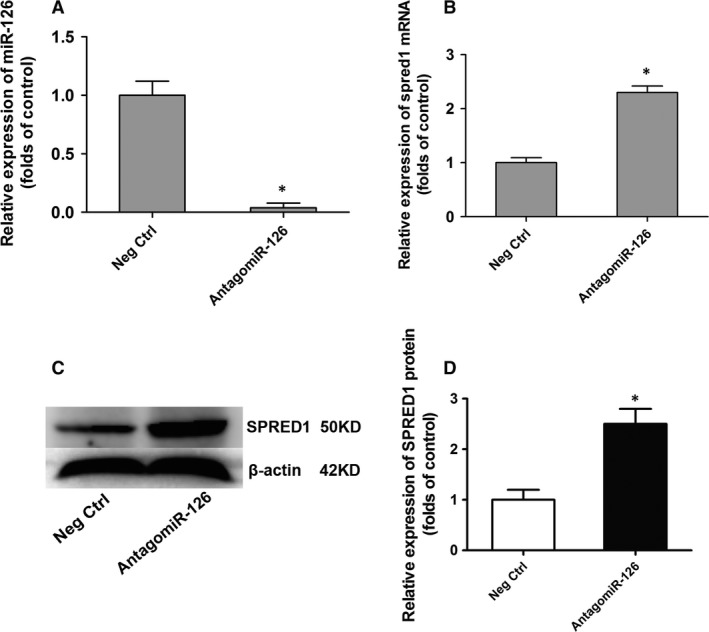
Inhibiting miR‐126 promoted *Spred1* expression in endothelial progenitor cells (EPCs). Bone marrow‐derived EPCs were transfected with 100 nM antagomiR‐126 after cultured for 7 days. And antagomiR negative control (Neg Ctrl) was used as control group. The expression of miR‐126 and mRNA of *Spred1* was tested by quantitative real‐time PCR after 24 hrs transfection. Relative expression of miR‐126 (**A**). Quantitative analysis of relative expression of *Spred1* mRNA (**B**). The protein of sprouty‐related EVH1 domain containing 1 (SPRED1) was tested by Western blotting after 48 hrs transfection. Examples of SPRED1 protein band in Western blotting (**C**). Quantitative analysis of relative expression of SPRED1 protein (**D**). *P <* *0.05, *versus* negative control (*n* = 3).

One of the most well‐studied, angiogenesis‐related target genes of miR‐126 is *Spred1*. To confirm that *Spred1* is a target of miR‐126 in EPCs, we used antagomiR‐126 (RIBOBIO) to silence miR‐126 *in vitro*; the antagomiR negative control (Neg Ctrl) (RIBOBIO) was used as the control. Bone marrow‐derived EPCs from NMI NET mice transfected for 24 hrs with 100 nM antagomiR‐126 had >100‐fold down‐regulated miR‐126 expression (*P *<* *0.05) (Fig. 4A). Next, we examined *Spred1* expression at transcriptional and post‐transcriptional levels after 24‐hr EPC transfection with 100 nM antagomiR‐126 and found 2.3‐fold increased relative expression of *Spred1* mRNA (*P *<* *0.05) (Fig. 4B). Moreover, the relative expression of SPRED1 protein was increased by 2.5‐fold after 48‐hr transfection with antagomiR‐126 (*P *<* *0.05), suggesting that *Spred1* is a target gene of miR‐126 in EPCs (Fig. 4C and D).

### TPPU inhibited *Spred1* expression in EPCs

**Figure 5:**
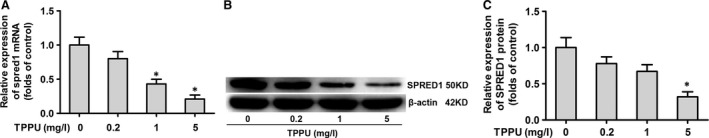
1‐trifluoromethoxyphenyl‐3‐(1‐propionylpiperidine‐4‐yl) urea (TPPU) inhibited *Spred1* expression in endothelial progenitor cells (EPCs). Bone marrow‐derived EPCs in the *in vivo* TPPU‐treated myocardial infarction (MI) ET group were isolated. The relative expression of *spred1* mRNA in EPCs was tested by quantitative real‐time PCR after 7‐day cultivation (**A**). Examples of sprouty‐related EVH1 domain containing 1 (SPRED1) protein band in Western blotting (**B**). Quantitative analysis of SPRED1 protein expression (**C**). *P <* *0.05, *versus* myocardial infarction with exercise training (MI ET) groups treated with 0 mg/l TPPU (*n* = 3).

EPCs were isolated and cultured from the *in vivo* TPPU‐treated MI ET groups. After 7 days of cultivation, we assayed the relative expression of SPRED1 mRNA and protein in the EPCs. *Spred1* expression was significantly decreased both at transcriptional and post‐transcriptional levels as the TPPU concentration increased. The MI ET mice treated with 5 mg/l TPPU had almost 4.0‐fold reduced *Spred1* mRNA expression when compared with the MI ET mice treated with 0 mg/l TPPU; SPRED1 protein expression was decreased by 3.1‐fold after treatment with 5 mg/l TPPU when compared with 0 mg/l TPPU (*P *<* *0.05) (Fig. 5A–C).

### TPPU modulated miR‐126 expression in EPCs by activating the AKT/GSK3β signalling pathway

**Figure 6:**
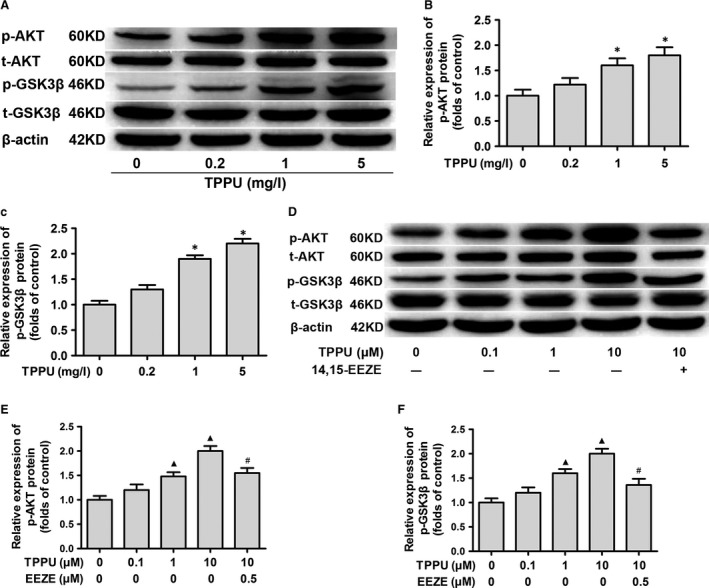
1‐trifluoromethoxyphenyl‐3‐(1‐propionylpiperidine‐4‐yl) urea (TPPU) activated the protein kinase B/glycogen synthase kinase 3β (AKT/GSK3β) signalling pathway. Western blot testing of relative expression of AKT, p‐AKT, GSK3β and p‐GSK3β in EPCs from TPPU‐treated myocardial infarction (MI) ET mice. Examples of protein bands are shown (**A**). Relative expression of p‐AKT (**B**) and p‐GSK3β (**C**). Western blot testing of relative expression of AKT, p‐AKT, GSK3β and p‐GSK3β in TPPU‐treated and TPPU plus 14,15‐EEZE‐treated EPCs from MI mice. Examples of protein bands are shown (**D**). Relative expression of p‐AKT (**E**) and p‐GSK3β (**F**). *P <* *0.05, *versus* 0 mg/l TPPU‐treated group. ^▲^ *P *<* *0.05, *versus* 0 μM TPPU group. ^#^ *P *<* *0.05, *versus* 10 μM TPPU group (*n* = 3).

Previously, we demonstrated that exercise promotes EPC functions by activating the AKT/GSK3β signalling pathway 5. Accordingly, we tested whether TPPU exerts its effects on EPCs by activating the AKT/GSK3β signalling pathway in this study. In the MI ET mice treated with 5 mg/l TPPU, the relative expression of p‐AKT and p‐GSK3β was increased by 1.8‐fold and 2.2‐fold, respectively, when compared with the MI ET mice treated with 0 mg/l TPPU (all, *P *<* *0.05) (Fig. 6A–C). *In vitro*, the AKT/GSK3β signalling pathway was also significantly activated after TPPU treatment. Similarly, p‐AKT and p‐GSK3β levels were markedly increased in EPCs after 24‐hr treatment with 10 μM TPPU; the addition of 14,15‐EEZE significantly reduced both p‐AKT and p‐GSK3β levels, indicating that TPPU exerts its function in EPCs by activating the AKT/GSK3β signalling pathway (Fig. 6D–F).

**Figure 7:**
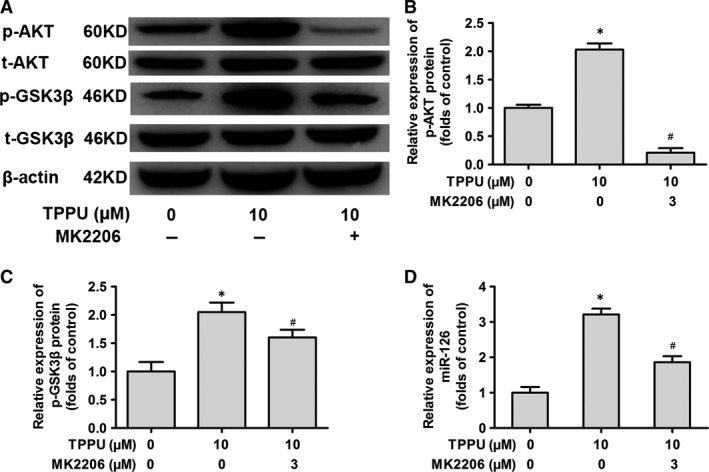
Blocking the protein kinase B/glycogen synthase kinase 3β (AKT/GSK3β) signalling pathway decreased 1‐trifluoromethoxyphenyl‐3‐(1‐propionylpiperidine‐4‐yl) urea (TPPU)‐induced miR‐126 expression. Western blot testing of relative expression of AKT, p‐AKT, GSK3β and p‐GSK3β in TPPU‐treated and TPPU plus MK‐2206‐treated EPCs from myocardial infarction (MI) ET mice. Examples of protein bands are shown (**A**). Relative expression of p‐AKT (**B**). Relative expression of p‐GSK3β (**C**). Quantitative real‐time PCR assay of relative expression of miR‐126 (**D**).*P <* *0.05, *versus* 0 μM TPPU‐treated group. ^#^ *P *<* *0.05, *versus* 10 μM TPPU‐treated group (*n* = 3).

To confirm the effects of the sEHI on miR‐126 *via* activation of the AKT/GSK3β signalling pathway, we used the AKT‐specific inhibitor MK‐2206 to block the AKT signalling pathway after treating EPCs with 10 μM TPPU *in vitro*. The relative expression of p‐AKT and p‐GSK3β was significantly reduced after 24‐hr treatment with 3 μM MK‐2206 when compared with TPPU treatment alone, indicating that MK‐2206 blocked the AKT/GSK3β signalling pathway (*P *<* *0.05) (Fig. 7A–C). Moreover, miR‐126 was down‐regulated 1.7‐fold after MK‐2206 intervention when compared with TPPU treatment alone (*P *<* *0.05) (Fig. 7D), suggesting that TPPU promotes miR‐126 expression partially by regulating the AKT/GSK3β signalling pathway.

## Discussion

**Figure 8:**
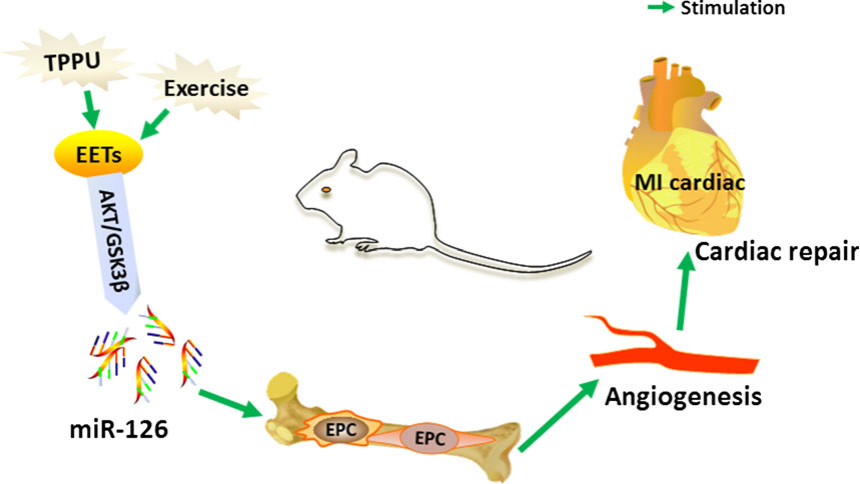
Diagrammatic illustration of the experiment. TPPU enhanced exercise‐induced EETs concentration to up‐regulate miR‐126 expression by activating AKT/GSK3β signalling pathway, and subsequently promoting EPC functions and angiogenesis in mice after MI. EETs: epoxyeicosatrienoic acids; EPC: endothelial progenitor cell; MI: myocardial infarction; TPPU: 1‐trifluoromethoxyphenyl‐3‐(1‐propionylpiperidine‐4‐yl) urea.

In this study, we report for the first time that exercise can increase serum EET levels, thereby promoting EPC functions in mice after MI. This result may be one reason for exercise‐induced angiogenesis around ischaemic areas in infarcted hearts. We also noted that exercise regulated miR‐126 expression and that TPPU could enhance the overexpression of miR‐126 and down‐regulate its target gene *Spred1* by elevating EET concentrations, which was associated with activation of the AKT/GSK3β signalling pathway (Fig. 8).

For the first time, we found that 4 weeks of exercise could significantly increase EET concentrations. As 14,15‐EET is mainly produced in the heart and exerts important cardioprotective function, we mainly focused on 14,15‐EET levels and not that of the other regioisomeric EETs. Although a growing number of studies have focused on the function of EETs and the related metabolites, we are the first to illustrate the relationship between exercise and EETs. As reported previously, exercise induces many mediators that exert important cardioprotective functions 29. Similarly, EETs are induced by exercise and may also be an exercise‐induced mediator for exerting cardioprotective properties after MI.

It has been widely demonstrated that EETs have multiple cardioprotective properties in cardiovascular diseases, including MI 9, 30, 31. However, studies have seldom focused on the effect of EETs on angiogenesis after MI 31. Previously, our group found that sEHIs could promote EPC function in patients with MI by increasing EET concentrations 11, which provided important evidence for the relationship between EETs and angiogenesis after MI. Interestingly, our previous work also demonstrated that exercise can markedly improve EPC function in mice after MI 5. Therefore, we speculated that EETs may also be involved in exercise improvement of EPC functions. In the present study, we mainly investigated whether and how EETs exert the cardioprotective function induced by exercise. Differing from our previous findings, the present findings demonstrate that TPPU, a novel sEHI that has more potent cardioprotective and pharmacological function than earlier sEHIs, improves EPC functions. More importantly, we also demonstrate that TPPU can further enhance exercise‐induced EPC function.

MiR‐126 is related to angiogenesis after MI and can also be up‐regulated by exercise. In the present study, we also found that exercise significantly increased miR‐126 expression in mice after MI. Partially in line with our findings, Uhlemann *et al*. 20 reported that a maximal symptom‐limited exercise test and 4 hrs of cycling resulted in a 2.1‐fold and 4.6‐fold increase, respectively, in circulating miR‐126 expression in healthy individuals. Chen *et al*. 23 reported that the transplantation of mesenchymal stem cells (MSCs) transfected with miR‐126 improved angiogenesis and cardiac function in the infarcted heart in MI mice. Although it has been well‐established by previous data that miR‐126 induces the promotion of angiogenesis and maintenance of vascular integrity, our study reports for the first time that exercise up‐regulates miR‐126 expression in EPCs to promote angiogenesis after MI. Furthermore, we also observed for the first time that TPPU further promotes the expression of miR‐126 in the exercised mouse model, and we illustrate the regulatory relationship between TPPU and miR‐126.

In the present study, we observed that TPPU activates the AKT/GSK3β signalling pathway in EPCs in mice after MI. Using MK‐2206 to block the AKT signalling pathway, we also show that TPPU induces miR‐126 expression at least partially by activating the AKT/GSK3β signalling pathway. Previously, we noted that exercise can activate the AKT/GSK3β signalling pathway, which is consistent with the findings of others 32. To date, the unique function of TPPU is to elevate EETs level by inhibiting sEH, and thereby, the mechanism of TPPU that promotes angiogenesis in our study is consistent with EETs’ to some extent. The most well‐studied mechanism of EETs‐induced angiogenesis is vascular endothelial growth factor (VEGF)‐dependent pathway, which includes both VEGF and VEGFR2‐independent mechanism that regulating angiogenesis. Yang *et al*. 33 not only demonstrated that the EETs promoted proliferative and angiogenic responses of cells to VEGF stimulation but also discussed that the phosphatidylinositol 3‐kinase (PI3K)/AKT and MAPK/ERK1/2 signalling pathways were involved in the EET‐mediated angiogenesis. Besides VEGF signalling pathway, Zhang *et al*. 34 found that EETs‐induced angiogenesis was also related to PI3K/AKT signalling *via* targeting in parallel with fibroblast growth factor 2 (FGF2) expression in human dermal microvascular endothelial cell, indicating that EETs stimulated angiogenesis *via* an EGF receptor‐dependent mechanism. Qu *et al*. 24 reported that elevated EET concentrations could protect against cerebral ischaemia/reperfusion injury, which is associated with AKT signalling pathway activation. Batchu *et al*. 35 found that EET‐mediated cardioprotection was involved in PI3K/AKT signalling pathway activation in a cardiac ischaemia/reperfusion mouse model. Overall, EETs perform their function *via* the AKT signalling pathway.

In our study, we demonstrate that the AKT/GSK3β signalling pathway is involved in TPPU‐regulated miR‐126 expression, thereby promoting EPC function. The most well‐studied target genes that related to angiogenesis of miR‐126 include *VEGFA*,* PIK3R2* and *SPRED1* as above mentioned 15, 16. Interestingly, previous study has also reported that miR‐126 regulates AKT signalling pathway by activating its target gene *PIK3R2*, another important target gene of miR‐126 that closely related to angiogenesis 36. In our present study, we mainly demonstrated *SPRED1* as the target gene of miR‐126 in MI mice model that underwent exercise owing to our preliminary experiment found it was the most significantly changed one in our studied model. Theoretically, there may exist positive feedback regulation between miR‐126 and AKT signalling pathway, and the expression of miR‐126 may be magnified markedly after AKT pathway activated. However, we did not observe a significantly amplified miR‐126 expression after TPPU treated, indicating the positive feedback in theory did not work, which might result from multiple and compensatory mechanism that induced by exercise and TPPU in organism.

Collectively, our findings provide new theoretical evidence for exercise‐based cardiac rehabilitation with the aim of improving cardiac rehabilitation recognition and enrolment. We also found that EETs are a potential exercise‐induced mediator and that TPPU, a novel sEHI that increases EET levels, may be a new agent for MI treatment.

## Limitations

There are limitations to the *in vivo* study that discussed the mechanisms of TPPU. We did not use sEH gene knockout mice to observe the effects of blocking sEH *in vivo*, which would have enabled more direct and authentic observation of the results. Besides, we did not test effects of exercise and TPPU on EPC functions in MI mice after miR‐126 knockout in this study. But exercise and TPPU at least partially exert their cardioprotection though regulating miR‐126 expression. Furthermore, exercise is a comprehensive programme that induces the activation of multiple organs and mechanisms in an organism. In the present study, we only tested EETs as a key exercise‐induced regulator for protecting ischaemic heart after MI. Exercise‐induced EPC function might also involve other factors and mechanisms, and there may be crosstalk between them. Therefore, further studies are needed to investigate this.

## Conflicts of interest

The authors reported no relationships that could be construed as a conflict of interest.
